# EGFR, VEGF, and angiogenesis promote the development of lipoma in the oral cavity

**DOI:** 10.1590/0103-6440202305117

**Published:** 2023-05-15

**Authors:** Weslay Rodrigues da Silva, Hévila de Figueiredo Pires, Glória Maria de França, Roseana de Almeida Freitas, Hannah Gil de Farias Morais, Hébel Cavalcanti Galvão

**Affiliations:** 1 Department of Oral Pathology, Federal University of Rio Grande do Norte, Natal, Brazil.

**Keywords:** mesenchymal tumor, oral lipoma, growth factor receptor, angiogenesis, angiogenic index

## Abstract

This study aimed to detect, quantify and compare the immunohistochemical expression of EGFR and VEGF and microvessel count (MVC) in oral lipomas, and to correlate the findings with clinical and morphological characteristics of the cases studied. The sample consisted of 54 oral lipomas (33 classic and 21 non-classic) and 23 normal adipose tissue specimens. Cytoplasmic and/or nuclear immunohistochemical staining of EGFR and VEGF was analyzed. The angiogenic index was determined by MVC. Cells were counted using the Image J® software. The Statistical Package for the Social Sciences was used for data analysis, adopting a level of significance of 5% for all statistical tests. A statistically significant difference in EGFR immunoexpression (*p*=0.047), especially, between classic lipomas and normal adipose tissue. There was a significant difference in MVC between non-classic lipomas and normal adipose tissue (*p*=0.022). In non-classic lipomas, only VEGF immunoexpression showed a significant moderate positive correlation (*r*=0.607, *p*=0.01) with MVC. In classic lipomas, the number of EGFR-immunostained adipocytes was directly proportional to the number of VEGF-positive cells, demonstrating a significant moderate positive correlation (*r*=0.566, p=0.005). The results suggest that EGFR, VEGF, and angiogenesis participate in the development of oral lipomas but are not primarily involved in the growth of these tumors.

## Resumo

Lipomas são as neoplasias mesenquimais benignas mais comuns, no entanto sua etiopatogenia ainda permanece desconhecida. Dessa forma, essa pesquisa teve como objetivo detectar, quantificar e comparar a expressão imunoistoquímica do EGFR, VEGF e contagem microvascular (MVC) dos lipomas orais, relacionando-os com as características clínicas e morfológicas dos casos estudados. A amostra foi composta por 54 lipomas orais (33 clássicos e 21 não clássicos) e 23 casos de tecido adiposo normal. A análise da expressão imunoistoquímica de EGFR e VEGF foi fundamentada na marcação citoplasmática e/ou nuclear. O índice angiogênico foi avaliado por meio da MVC. A contagem de células foi realizada utilizando *software* IMAGE J®. Os dados obtidos foram analisados no software Statistical Package for Social Science. O nível se significância de 5% foi adotado para os testes estatístico. A análise da imunoexpressão das proteínas revelou para o EGFR diferença estatisticamente significativa (*p*=0,041) entre o lipoma clássico e o tecido adiposo normal. Houve diferença significativa na MVC entre lipomas não clássicos e tecido adiposo normal (*p*=0,022). Nos lipomas não clássicos, apenas a imunoexpressão de VEGF apresentou correlação do tipo moderada, positiva e significativa (r=0,607; *p*=0,010) em relação a MVC. Ademais, nos lipomas clássicos foi percebido que os adipócitos imunomarcados para EGFR estiveram diretamente proporcionais a imunoexpressão de VEGF, apresentando correlação do tipo moderada, positiva e estatisticamente significativa (r=0,566; *p* = 0,005). Com base nos resultados, pode-se sugerir que o EGFR, VEGFR e MCV participam do desenvolvimento nos lipomas orais, contudo, não estão primariamente envolvidos no crescimento tumoral dessas neoplasias.

## Introduction

Lipomas are the most common benign mesenchymal tumors.^1^ ^)-(^ ^3^ These tumors have a predilection for the trunk, shoulder, neck, and axilla. Lipomas in the head and neck region account for 20% of all cases, while lipomas in the maxillofacial region are uncommon. The incidence of oral lipomas ranges from 1 to 4%, with no sex preference of these and occurrence over a wide age range.^3^ ^)-(^ ^6^

Clinically, oral lipomas are generally painless and manifest as a sessile or pedunculated nodule of soft consistency and slow growth.^3^ ^),(^ ^4^ ^),(^ ^7^ The cheek mucosa is the most affected site, followed by the tongue and lip.^3^ ^)-(^ ^5^ Although rare, lipomas can occur in gnathic bones, accounting for 3% of all intraosseous lipomas in the body.^4^

Histologically, lipomas are composed of mature adipose tissue surrounded by a fibrous connective tissue capsule from which septa project that divide the tumor into lobules. There are several histological variants of lipoma. The most prevalent variants in the oral cavity are classic lipoma, fibrolipoma, intramuscular lipoma, sialolipoma, chondrolipoma, and spindle cell/pleomorphic lipoma.^3^ ^)-(^ ^5^

Different families of receptors and growth factors are involved in the growth of normal and neoplastic tissues. Among these receptors, the epidermal growth factor receptor (EGFR) plays important role in cell proliferation and migration.^8^ ^),(^ ^9^ In addition, EGFR participates in angiogenesis, inducing increased expression of vascular endothelial growth factor (VEGF).^10^ ^),(^ ^11^

Most studies have investigated the association between EGFR and VEGF in malignant tumors,^12^ ^)-(^ ^14^ while little is known about the role of these proteins in benign mesenchymal tumors such as lipomas. Therefore, the aim of this study was to detect, quantify and compare the immunohistochemical expression of EGFR and VEGF and microvessel count (MVC) in oral lipomas, and to correlate the findings with clinical and morphological characteristics of the cases studied.

## Material and methods

### Study design

The Ethics Committee of the Federal University of Rio Grande do Norte (UFRN) approved the study (Approval number 4.426.500). This was a retrospective, observational, cross-sectional study that analyzed and quantified the immunohistochemical expression of EGFR and VEGF in oral lipoma cases stored at a referral pathological anatomy service in northeastern Brazil.

### Samples

Intentional, non-probability sampling was used. The sample consisted of 54 cases of different morphological types of oral lipoma and 23 normal adipose tissue specimens as a control group. Only cases whose medical records contained all data necessary for the clinical study were included. Specimens showing flaws or problems during cutting and processing that impaired the morphological and immunohistochemical analyses were excluded. For the control group, the cases of normal adipose tissue included in this study came from bichectomy surgeries, without surrounding inflammatory infiltrate or any other lesion, checked in H&E in light microscopy.

### Morphological analysis

The specimens were stained with hematoxylin and eosin, cut into 5-µm sections, and examined under a light microscope (Olympus CX31, Olympus Japan Co., Tokyo, Japan) to confirm the histopathological diagnosis of each lesion.

Data regarding the presence of a capsule and inflammatory infiltrate were collected. The histological variants of lipoma were classified according to the method adapted from Juliasse et al.^4^ and Linares et al.^3^, based on the most prevalent variants, into classic lipoma and non-classic lipoma (fibrolipoma, intramuscular lipoma, sialolipoma, chondrolipoma, and spindle cell/pleomorphic lipoma). For confirmation of the diagnosis of spindle cell/pleomorphic lipoma, 3-µm histological sections mounted on silanized slides were submitted to immunohistochemistry using the following antibodies: anti-S100 (polyclonal, diluted 1:10,000), CD34 (clone QBEnd10, diluted 1:50), and specific muscle actin (clone HHF35, diluted 1:800).

### Immunohistochemical analysis

For immunohistochemical analysis, 3-µm-thick sections were mounted on slides coated with organosilane (3-aminopropyltriethoxy silane; Sigma Chemical Co., St. Louis, MO, USA). These histological sections were submitted to immunoperoxidase staining by the dextran polymer technique using anti-EGFR (SP9, 1:200, 60’; Spring Bioscience, Pleasanton, CA, USA) and anti-VEGF antibodies (Cc-7269, 1:200, overnight; Abcam, Cambridge, United Kingdom) and diaminobenzidine as chromogen. As a positive control, histological sections of oral squamous cell carcinoma were used for EGFR and endothelial cells of the blood vessel wall for VEGF. In the negative control, the primary antibody was replaced with 1% bovine serum albumin in a buffer solution.

After processing the histological sections and immunohistochemistry, each specimen was analyzed under a light microscope by an examiner previously trained by an experienced pathologist. EGFT and VEGF were analyzed quantitatively using an adaptation of the method described by Lee et al.^2^. For both markers, cells with brown staining in the cytoplasmic membrane and/or nucleus were classified as positive, regardless of intensity. The cells were counted using the Image J® software. Ten fields of highest immunoreactivity were identified at 100x magnification. These fields were photographed at 400x magnification with a digital camera coupled to the microscope. The images obtained were transferred to a computer using the Infinity Analyze software and the cells were then counted. All cells (positive and negative) were counted in each photographed field and the percentage of positive cells was calculated for each case.

The angiogenic index was determined by MVC. Using an adaptation of the method described by Maeda et al.^15^, 10 fields of the highest immunoreactivity to the VEGF antibody were identified at 100x magnification and then photographed at 400x. The number of microvessels was counted in each field and the values obtained were summed to obtain the total MVC. Finally, the mean number of microvessels per field was calculated for each case. Individual immunopositive cells as well as clusters of immunopositive cells were considered to be microvessels, regardless of the presence of a formed lumen.

### Statistical analysis

The results were analyzed using the IBM SPSS Statistics 20.0 program (IBM Cop., Armonk, NY, USA). Descriptive statistics were used for the characterization of the sample. The distribution of the data was analyzed by the Shapiro-Wilk test. The Kruskal-Wallis test was used to determine significant differences in the quantitative variables (EGFR, VEGF, and MVC) between the three groups (classic lipomas, non-classic lipomas, and normal adipose tissue). The adjusted Dunn post-test was applied to verify the significant difference between groups. The correlation between the quantitative variables was evaluated using Spearman’s test. A level of significance of 5% (*p*≤0.05) was adopted for all statistical tests.

## Results

### Clinical and morphological data

The sample consisted of 33 (61.1%) classic lipomas and 21 (38.9%) non-classic lipomas (8 fibrolipomas, 1 intramuscular lipoma, 7 sialolipomas, 2 chondrolipomas, and 3 spindles cell/pleomorphic lipomas). Twenty-three normal adipose tissue specimens, including 10 buccal fat pad specimens, were used as control. Women were the most affected, with a female-to-male ratio of 1.57:1. The mean age was 57.1 ± 15.9 years.

The time to diagnosis ranged from 2 months to 15 years, with a mean of 3.2 years. Forty-eight (88.6%) cases were asymptomatic. All tumors evaluated showed exophytic growth and the mean tumor size was 2 ± 1.7 cm (range 0.3 to 6 cm). The sites most frequently affected by the tumors were the cheek mucosa (20.3%) and lip (20.3%). Table 1 shows the clinicopathological data.

### Immunohistochemical analysis

Some degree of EGFR and VEGF immunostaining was observed in all cases and controls. EGFR immunopositivity was detected in 56.6% of cells in classic and non-classic lipomas, while this percentage was 37.5% in normal adipose tissue. Neoplastic and normal adipocytes exhibited membrane and nuclear expression of this protein. Regarding VEGF, 62.2% of cells in lipomas were immunoreactive, while 47.6% of adipocytes in normal adipose tissue expressed this growth factor. Nuclear expression of this protein was found in neoplastic and normal adipocytes.

**Figure 1:**
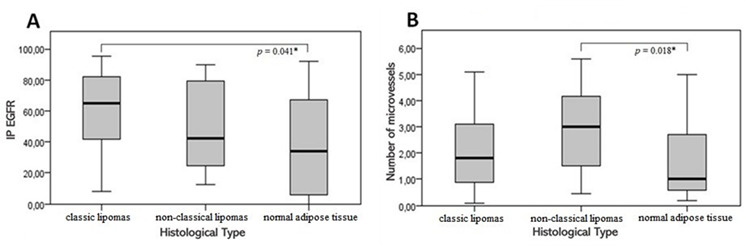
A) Box-plot relating to immunostaining for EGFR, according to lipoma and normal adipose tissue groups (Dunn post hoc test). B) Blox-plot relative to the angiogenic index, according to the lipoma and normal adipose tissue groups (Dunn post hoc test).

**Figure 2:**
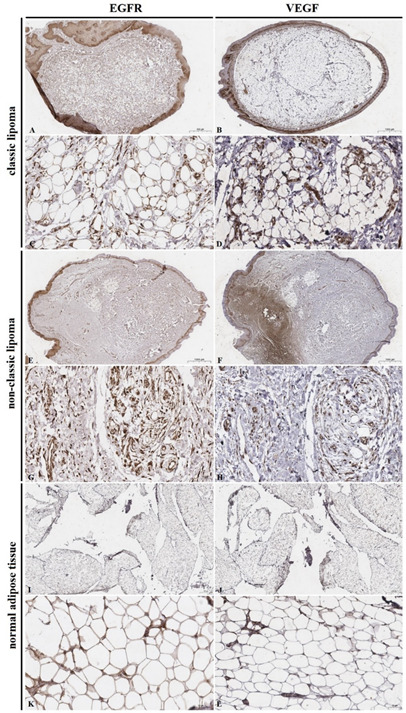
(A, C, E, G, I, K) Immunoexpression of nuclear and membrane EGFR in classic lipomas, non-classic lipomas, and normal adipose tissue. (B, D, F, H, I, L) Nuclear immunoexpression of VEGF in classic lipomas, non-classic lipomas, and normal adipose tissue. (Scale bar 50-1000 μm).

EGFR was overexpressed in lipomas, especially in classic lipomas with a median score of 65.0, followed by non-classic lipomas (median = 42.4) and normal adipose tissue (median = 33.8) (Table 2). In addition, there was a statistically significant difference in EGFR expression (*p*=0.047, table 2), especially, between classic lipomas and normal adipose tissue by Dunn’s post hoc test (*p*=0.041, Figure 1). Immunoreactivity to VEGF was higher in classic lipomas, followed by non-classic lipomas and normal adipose tissue (median scores of 64.3, 57.7, and 52.8, respectively). Evaluation of the VEGF-immunostained specimens revealed no significant difference in the immunoexpression of this protein between lipomas and normal adipose tissue (Table 2 and Figure 2).

**Table 1:** Clinical and morphological aspects of oral cavity lipomas.

Clinical-morphological parameters	Classic lipomas		Non-classical lipomas		Total	
	N	%	N	%	N	%
Gender						
Female	20	60.6	13	61.9	33	61.1
Male	13	39.4	8	38.1	21	38.9
Total	33	100	21	100	54	100
Race						
Leukoderma	8	24.2	7	33.3	15	27.7
Melanoderma	7	21.2	3	14.2	10	18.5
Pheoderm	18	54.6	11	52.5	29	53.8
Total	33	100	21	100	54	100
Symptomatology						
Yes	1	3.0	5	23.8	6	11.4
No	32	97.0	16	76.2	48	88.6
Total	33	100	21	100	54	100
Localization						
Cheek mucosa	6	18.3	5	23.8	11	20.3
LIP	9	27.3	2	9.5	11	20.3
Alveolar ridge	2	6.0	1	4.9	3	5.5
Tongue	3	9.0	5	23.8	8	14.8
Palate	4	12.1	2	9.5	6	11.4
Floor	1	3.0	2	9.5	3	5.5
Retromolar trigone	2	6.0	2	9.5	4	7.4
Vestibule background	6	18.3	2	9.5	8	14.8
Total	33	100	21	100	54	100
Clinical aspect ^#^						
Nodule	19	61.3	11	84.6	30	55.5
Tumor	12	38.7	2	15.4	14	25.9
Total	31	100	13	100	44	81.4
Implantation						
Sessile	19	57.6	12	57.1	31	57.4
Pedunculated	14	42.4	9	42.9	23	42.6
Total	33	100	21	100	54	100
Lesion color						
Normochromic	16	48.4	10	47.7	26	48.1
Yellow	11	33.3	7	33.3	18	33.3
Red	6	18.3	2	9.5	8	14.8
Blue	0	0.0	2	9.5	2	3.8
Total	33	100	21	100	54	100
Consistency						
Soft	22	66.7	13	61.9	35	64.8
Fibrous	11	33.3	8	38.1	19	35.2
Total	33	100	21	100	54	100
Inflammatory infiltrate						
Yes	19	57.6	3	14.2	22	40.7
No	14	42.4	18	85.8	32	59.3
Total	33	100	21	100	54	100
Capsule						
Yes	23	69.3	7	33.3	30	55.5
No	10	30.3	14	66.7	24	44.5
Total	33	100	21	100	54	100

# Missing data from biopsy records

**Table 2:** Percentage of EGFR and VEGF immunoexpression in adipocytes and vessel count in lipomas and normal adipose tissue.

Histological type	n	EGFR % Median (Q25-Q75)	*p*	VEGF Adipócitos Median (Q25-Q75)	*p*	MVC Median (Q25-Q75)	*p*
CL	33	65.0 (41.6 - 82.0)	0.047*	64.3 (44.0 - 90.4)	0.142	1.8 (0.9 - 3.1)	0.022*
NCL	21	42.4 (24.9 - 79.5)		57.7 (43.1 - 78.2)		3.0 (1.5 - 4.1)	
NAT	23	33.8 (5.7 - 67.1)		52.8 (19.8 - 74.0)		1.0 (0.6 - 2.6)	

CL=classic lipoma; NCL=non-classical lipoma; NAT=normal adipose tissue; *=Statistically significant difference for the Kruskal-Wallis test.

**Figure 3:**
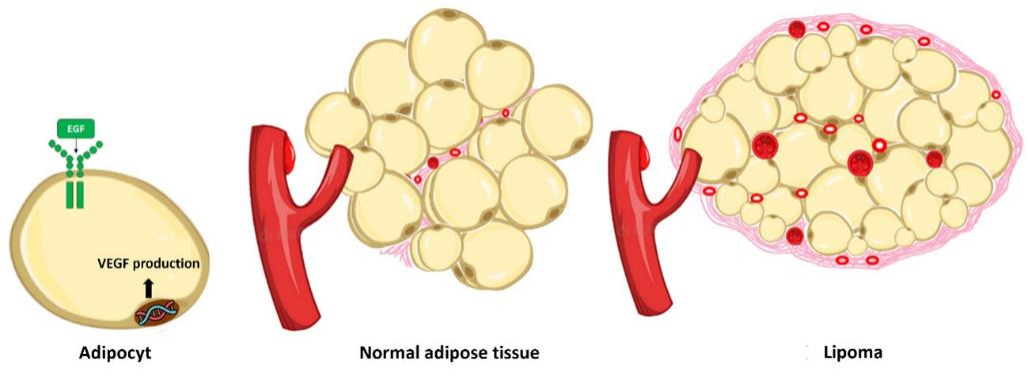
Representation of the difference in vascularization between lipomas and normal adipose tissue.

The angiogenic index was determined by MVC using an anti-VEFG antibody. Immunoexpression of this protein was observed in endothelial cells of vessels with a formed lumen, in endothelial cell clusters without a distinct vascular lumen, and in individual cells. The median number of microvessels was 1.8 in the group of classic lipomas and 3.0 in non-classic lipomas (2.8, 3.0, 2.3, and 4.2 in fibrolipoma, sialolipoma, chondrolipoma, and spindle cell/pleomorphic lipoma, respectively), while this number was 1.0 in normal adipose tissue (Table 2). There was a significant difference in MVC (*p* = 0.022, table 2) between groups, especially between non-classic lipomas and normal adipose tissue by Dunn’s post hoc test (*p* = 0.018, Figures 1 and 3).

In classic lipomas, the number of EGFR-immunostained adipocytes was directly proportional to the number of VEGF-positive cells, demonstrating a significant moderate positive correlation (*r*=0.566, *p*=0.005), although MVC was not significantly correlated with EGFR immunoexpression in this tumor. In non-classic lipomas, only VEGF immunoexpression was directly proportional to MVC, with a significant moderate positive correlation (*r*=0.607, *p*=0.01). Furthermore, there was no significant correlation between tumor size and the antibodies evaluated (Table 3).

**Table 3:** Correlation between the studied antibodies, vessels and size in cm.

Variables	Lipomas				Normal adipose tissue	
	Classics		Non-classics			
	*r*	*p*	*r*	*p*	*r*	*p*
EGFR % X VEGF %	0.566	0.005*	-0.151	0.658	-0.341	0.213
Size X EGFR%	-0.131	0.542	-0.190	0.534	-0.311	0.381
Size X VEGF%	-0.272	0.198	0.276	0.339	0.131	0.805
EGFR% X MVC	0.199	0.387	-0.242	0.473	0.439	0.102
VEGF % X MVC	0.101	0.616	0.607	0.010*	-0.162	0.549
Size X MVC	-0.032	0.888	-0.381	0.179	-0.655	0.158

*=significant results for the Spearman correlation test; *r*=correlation coefficient.

## Discussion

The present study used 54 oral lipoma specimens to investigate the immunoexpression of EGFR and VEGF proteins involved in tumor proliferation, growth, and angiogenesis. Our results suggest that these proteins are associated with the development of these tumors. To our knowledge, there are a few studies^2^ that investigated the participation of these proteins and the angiogenic index in the development of oral lipomas.

Different families of growth factors and their respective receptors are involved in cell proliferation, differentiation, angiogenesis, and tumorigenesis.^8^ ^),(^ ^9^ ^),(^ ^16^ ^)(^ ^17)^ ^-(^ ^18^ Within this context, EGFR and VEGF have been studied mainly in malignant tumors of epithelial origin.^13^ ^),(^ ^19^ Although scarce, some studies have investigated these proteins in benign mesenchymal tumors such as hemangiomas and leiomyomas.^20^ ^),(^ ^21^

EGFR was overexpressed in the present study, with the observation of immunoreactivity in 56.6% of the tumor cells analyzed. Lee et al.^2^ evaluated the immunoexpression of EGFR in 20 cases of lipomas affecting different sites of the body and suggested the participation of this protein in neoplastic growth. The authors detected immunoreactivity in 30% of their sample, while in our study all cases displayed some degree of immunoreactivity.

In the present study, EGFR immunostaining was higher in lipomas than in normal adipose tissue (37.5%) and this difference was statistically significant. This result may be related to the characteristics of normal adipose tissue that has a lower proliferation rate than lipomas, which are neoplastic.^11^ ^),(^ ^19^ ^),(^ ^22^

Although EGFR plays important roles in cell proliferation, differentiation, and migration,^8^ ^),(^ ^9^ ^),(^ ^18^ the present study did not find a significant correlation between EGFR immunoexpression and the size of the tumors studied, in agreement with the findings of Brahim et al.^23^. This lack of correlation may be due to the benign nature of oral lipomas since EGFR is overexpressed mainly in sarcomas.^24^

The role of EGFR in mesenchymal tumors is little explored. Dobashi et al.^24^ evaluated changes and activation of EGFR and EGFR-mediated signaling cascade in 31 sarcomas and malignant bone tumors compared to 8 benign lesions of mesenchymal origin by immunohistochemical analysis, FISH, Western blot, and nucleotide analysis. EGFR was overexpressed in 22.6% of sarcomas but not in any of the benign lesions. The authors suggested that persistent activation of Stat-3 may be a critical event due to the overexpression of EGFR and that EGFR mutation may activate other signaling pathways.

The EGFR signaling pathways do not only induce cell proliferation and inhibit apoptosis but are also associated with angiogenesis, with the development of an appropriate vascular network being necessary for tumor growth, invasion, and metastasis. Thus, the interaction of EGF with its ligands, such as EGFR and TGF-α, induces VEGF expression through the PI3K/AKT and RAS/RAF/ERK pathways, in addition to the upregulation of HIF-1α.^10^ ^),(^ ^25^ ^)(^ ^26^ ^)-(^ ^27^ The present study revealed a significant moderate positive correlation between EGFR and VEGF demonstrating the presence of such correlation in mesenchymal tumors such as oral lipomas.

Once secreted by tumor cells, VEGF interacts with endothelial cells and induces angiogenesis, contributing to tumor development.^25^ ^),(^ ^18^ In the present study, VEGF was detected in 62.2% of the neoplastic cells evaluated, a higher rate of immunopositivity than that found in the control group (47.6%). In contrast, Lee et al.^2^, evaluating the immunoexpression of VEGF in lipomas affecting different sites of the body, found immunoreactivity in 90% of their sample. Since lipomas at extraoral sites have a longer average evolution time than oral lipomas,^28^ ^),(^ ^29^ we believe that this difference in immunoexpression may be related to the location of the tumor. Lee et al.^2^ showed that, unlike intraoral tumors, tumors at extraoral sites had a longer evolution time and possibly a more developed vascular network.

The capacity of lipomas to trigger an angiogenic response is an essential step in the growth of these tumors.^2^ Nevertheless, this study found no significant correlation between the size of lipomas and VEGF immunoexpression.

The immunohistochemical expression of VEGF is generally lower in normal tissue than in neoplastic tissue because of the need of malignant tumors for nutrition and dissemination.^25^ ^),(^ ^30^ Although in the present study, VEGF immunoreactivity was higher in neoplastic tissue than in normal tissue, the difference was not statistically significant, possibly because benign neoplasms and not malignant tumors were compared to normal tissue.

There are several approaches other than analysis of the expression of growth factors related to vascular development to study angiogenesis.^10^ ^),(^ ^25^ ^)-(^ ^27^ ^),^ One possibility is the measurement of the angiogenic index.^15^ ^),(^ ^31^ Studies suggest that an increase in the density or number of vessels is related to the prognosis of malignant tumors of mesenchymal and epithelial origin.^32^ ^),(^ ^33^

Microvessel density is believed to be lower in normal tissue compared to neoplastic tissue and that it is related to the histological grade of the tumor.^33^ In the present study, the median MVC was higher in non-classic lipomas compared to classic lipomas and normal adipose tissue. We believe that this greater angiogenic potential of the non-classic types may be related to the particularities of the tumor stroma, which is more abundant and thus favors vascular development.

The present study found a significant difference in MVC between non-classic lipomas and normal adipose tissue. Considering the importance of VEGF as an angiogenic inducer, including in tumors, and its capacity to trigger an increase in microvessel density, including endothelial proliferation, the formation of new vessels, and the recruitment of perivascular elements,^33^ this study evaluated the correlation between VEGF and MVC and found a statistically significant correlation between VEGF immunoexpression and the angiogenic index. Koh et al.^34^ reported a similar correlation between VEGF and microvessel density in Hodgkin’s lymphomas.

In conclusion, the results of the present study demonstrate the participation of EGFR and VEGF in the development of oral lipomas. In addition, the larger number of microvessels observed in non-classic lipomas suggests a role of angiogenesis in the development of these tumors. The lack of a significant correlation between EGFR, VEGF, and MVC with tumor size suggests that neither these proteins nor angiogenesis are primarily involved in tumor growth.
